# A Novel Curriculum to Optimize Emergency Medicine Residents’ Exposure to Pediatrics

**DOI:** 10.5811/westjem.2016.10.31248

**Published:** 2016-11-15

**Authors:** Chris Merritt, Sarah A. Gaines, Jessica Smith, Sally A. Santen

**Affiliations:** * Alpert Medical School of Brown University, Rhode Island Hospital/Hasbro Children’s Hospital, Department of Emergency Medicine, Section of Pediatric Emergency Medicine, Providence, Rhode Island; † Alpert Medical School of Brown University, Rhode Island Hospital, Department of Emergency Medicine, Providence, Rhode Island; ‡ University of Michigan Medical School, Department of Emergency Medicine, Ann Arbor, Michigan

## BACKGROUND

Emergency medicine (EM) residency graduates have a profound impact on the quality of pediatric emergency care. Consequently, residency training programs must provide broad clinical training in both adult and pediatric emergency medicine (PEM).^1^–^6^ The teaching of pediatrics to EM residents has historically included an inpatient “ward” rotation.^7^ Inpatient rotations are provided to help EM residents understand the experience of the hospitalized child, yet the educational benefit for EM trainees is not valued as much as educational experiences in the setting of the emergency department (ED).

Designing a high-yield pediatric experience that allows EM residents to understand the progression of common illnesses and anticipate the medical and psychosocial needs of hospitalized children remains a challenge. Awareness of this need led us to develop a novel curriculum in pediatrics for EM residents – Pediatric Emergency Medicine with Follow-Up (PEMFU). The goal of this curriculum is to prepare EM residents to provide pediatric emergency care via a situated experience in pediatric medicine.

## OBJECTIVE

Our goal is to describe the development of a novel curriculum for teaching and learning pediatric medicine in an EM residency program based on an assessment of need and structured around the conceptual framework of situated learning. We also describe the implementation of this curriculum within a single EM residency, and report early outcomes.

## CURRICULAR DESIGN

### Needs Assessment - The Case for a New Approach

After feedback from educators, residents, and medical directorship suggested that educational needs were unmet under the traditional pediatric ward rotation format, the ward rotation was withdrawn from our residency curriculum. In its stead, PEMFU was designed using the six-step curriculum development framework developed by Kern beginning with general and targeted needs assessments.^8^

Through focus groups, anonymous surveys of current and former residents, and numerous discussions, we identified five key aspects of the inpatient pediatric rotation important to the development of pediatric competence within EM residency that may be unavailable in other venues.

- Assessed Need 1: Inpatient rotations foster interactions with patients and families, and experience with the pediatric physical examination. The new curriculum (PEMFU) would need to foster developmentally-appropriate and family-centered practice via experience caring for children and families.
- Assessed Need 2: EM residents must understand and anticipate the needs of hospitalized pediatric patients. PEMFU would need to allow trainees to understand the continuum of pediatric illness/injury and the evidence-based management and disposition of hospitalized children.
- Assessed Need 3: EM residents risk losing the collegial relationships formed by working side by side with inpatient providers and consultants. At a time when more hospitalized patients make their way through the ED, PEMFU would need to address this unintended social-professional consequence, and continue to allow EM residents to develop working relationships with other healthcare professionals.
- Assessed Need 4: There should be an emphasis on the critical importance of lifelong, self-directed learning to the practice of medicine. PEMFU would need to allow EM residents to reinforce the skills critical to lifelong self-directed learning in EM.
- Assessed Need 5: While an understanding of inpatient pediatrics remains important to the educational development of EM residents, adult learners learn better via participating in a social and professional setting that more closely mirrors their future practice. This concept of *situated learning*^9^ was vital to the development of the PEMFU curriculum. PEMFU would need to allow EM residents to develop skill and expertise in pediatric care in the context of the practice of emergency medicine.

Learning objectives, mapped to the needs assessment, were developed to guide curriculum development under the aegis of a single broad goal of learning to provide excellent pediatric emergency care (Table 1). A logic model was developed to guide the education strategies employed and to direct program evaluation (Appendix).^10^

PEMFU was implemented in the 2014–2015 academic year; 12 second-year resident learners rotated in the pilot season. Eight components comprise the curriculum (Table 2).

The bulk of the clinical experience takes place from caring for patients in the ED – the social-professional environment most meaningful to future emergency physicians.^11^–^14^ Core to this innovation is that residents continue to follow the course of all patients they admit to the hospital. Residents regularly visit their patients and families in the hospital, read daily progress and consultation notes, follow up on test results, and interact with the inpatient teams to discuss the ongoing care of these admitted patients. Free of the administrative burden of the ward teams, EM residents observe the longitudinal course of illness and the experiences of hospitalized children and families.

Additionally, residents round once weekly on the wards with a PEM subspecialist experienced in inpatient medicine, visiting the bedside, engaging in discussion of diagnosis and treatment as well as the psychosocial experiences of hospitalization. Residents follow up by phone with a smaller subset of discharged patients. Dedicated time has been built into residents’ schedules to accommodate these follow-up activities.

## IMPACT AND EFFECTIVENESS

As part of programmatic assessment,^15^ we employed a series of assessment components from a variety of raters to provide feedback to learners and guide continuous quality improvement for the experience.

Clinical Assessment: Clinical faculty who supervise EM residents in the ED assess resident performance and provide formative feedback, recording this assessment in an online form. These assessments collectively contribute to the residents’ summative assessment.

Direct Observation: One faculty member performs a monthly 2–3 hour direct observation session during an ED shift for each resident. Structured formative feedback is provided covering important skills of history-taking, age-appropriate pediatric physical examination, diagnostic decision-making, and a developmentally-appropriate approach to children. A self-reflection exercise is built into the feedback process, encouraging reflection for ongoing professional identity formation.

Summary Assessment: A summary assessment is completed by the course director, assessing the core elements of the curriculum, including the resident’s written work product, interactions during ward rounds and teaching sessions, and group-sourced feedback from faculty, nursing, and other staff. PEMFU provides important insight into the progression of EM residents through the EM Milestones,^16^ particularly Milestone 7 (Disposition) and Milestone 17, in which residents must demonstrate “awareness of and responsiveness to the larger context and system of health care.”

### Evaluation and Feedback from Stakeholders

Reactions to PEMFU have been favorable from faculty, residents, and families. Parents, in particular, appreciate that emergency providers are committed to following up with each patient, even when they are no longer specifically responsible for their care. Specific strengths cited by residents include developing close working relationships with the clinical faculty mentors, as well as increased access to pediatric colleagues, and increased individualized interactive instruction.

**Figure 1:**
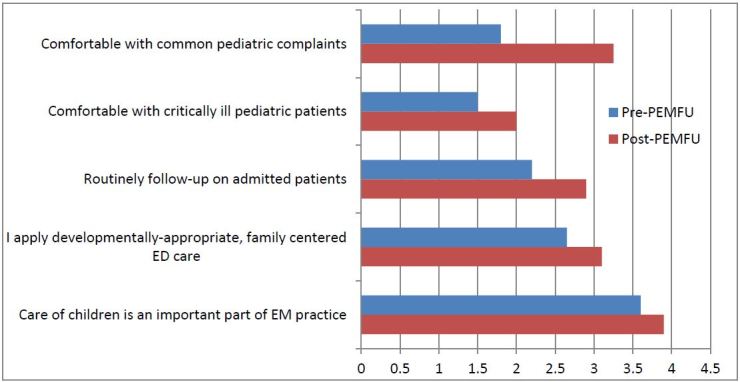
Residents’ reported attitudes and behaviors before and after participation in the PEMFU curriculum. Twelve residents responded to this retrospective post-then-pre survey, asking them to consider their current (post-PEMFU) attitudes and behaviors, and then asked to consider their attitudes and behaviors before participating in the curriculum (pre-PEMFU). Attitudes and behaviors were measured by assessing agreement with a series of statements on a scale from 1 (Strongly Disagree) to 5 (Strongly Agree). *PEMFU,* pediatric emergency medicine with follow up; *ED*, emergency department; *EM* emergency medicine

Following Kirkpatrick’s evaluation model,^17^ reactions to the program are regularly solicited from residents completing the rotation, using face-to-face debriefing and online evaluation forms. A retrospective post-then-pre survey was performed to gauge the curriculum’s impact on residents’ attitudes and self-reported behaviors (Figure 1). In addition, 11 of 12 residents responded with “agree” or “strongly agree” with the statement: *“The PEM-FU rotation has led to improved patient care and outcomes for my ED patients.”* This evaluation was performed in the course of educational quality improvement, and was exempted from review by the medical center’s institutional review board.

Recommendations for improvement have related to specific clinical content: interpreting pediatric radiographic studies, exposure to pediatric critical care, the psychomotor aspects of interacting with children (especially infants and toddlers), and concerns regarding the seasonal nature of disease processes seen in the pediatric ED.

A number of constraints make higher-level evaluation of educational outcomes challenging. In-training examinations, based on the American Board of Emergency Medicine Core Content, do not distinguish pediatrics from other components of EM practice; pediatric-specific knowledge measurements are difficult to ascertain. Whether this novel curriculum will have effects on important clinical outcomes in the longer term remains to be seen. Further, the literature on pediatric training within EM residency consists mainly of content recommendations; specific curricular recommendations are lacking, making comparisons between curricular approaches challenging.^2^

Teaching the PEMFU program is effort-intensive and requires administrative support to allow time for weekly attending rounds, observation sessions, and ad hoc teaching sessions. In our model, a single PEM specialist has responsibility for supervision and planning, though this could conceivably be shared among several faculty.

## DISCUSSION

This curriculum represents an example of *situated learning,* in which residents learn by working clinically in the environment where their knowledge and skills will be put to use in their future professional practice (i.e. in the ED caring for ill and injured children), rather than extrapolating from a foreign learning environment. This is thought to contribute to deeper and more meaningful learning.^18^ Through the use of mentored learning, PEMFU employs the methods described within a *cognitive apprenticeship*; supervising faculty provide contextualized support while supervising clinical care through role modeling, coaching, and articulation techniques.^19^,^20^

**Figure 2:**
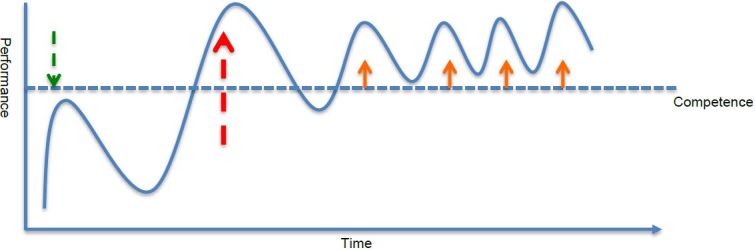
The “Bolus and Drip” Model - A framework for understanding pediatric emergency medicine (PEM) experience curves as experienced by EM residents in a four-year EM residency program. There are month-long PEM experiences (boluses) in each of the first two years of residency (dashed arrows), of which the described curriculum constitutes the second “bolus” – the longer dashed arrow. This is followed by a series of PEM shifts, interspersed among the remainder of the four-year residency experience (the “drip”, solid arrows), meant to provide PEM refreshers, and to simulate the frequency with which a typical general EM provider can expect to manage pediatric patients.

We know that knowledge and skills, if not used regularly, do not last. Pusic and colleagues have described “experience curves” chronicling the process of knowledge and skill accrual, followed by decay, with return to competence via interval training or experience.^21^,^22^ The PEMFU experience fits into a longitudinal PEM framework within our EM residency program intended to minimize this decay. Likened to a “bolus and drip,” there are two intensive pediatric experiences (“boluses”) in the first two years (PEMFU comprises the second “bolus”), followed in the third and fourth years by a “drip” of PEM shifts interspersed into the longitudinal clinical experience. We believe that this PEM curriculum, augmented with refresher experiences over the subsequent two years (Figure 2), will help learners achieve and maintain competence.

## CONCLUSION

We have developed a novel educational method, with dedicated pediatric ED time, deliberate patient follow up, ward rounds, discussion of focused pediatric topics, and direct observation. This model could be customized to fit a variety of educational settings in pediatric or adult medicine training for emergency physicians. We believe that this novel curriculum represents one model for integrating knowledge of pediatric illness and injury – acute and longitudinal – into the emergency medicine residency education paradigm.

## Supplementary Information



## Figures and Tables

**Table 1:** Pediatric emergency medicine with follow-up (PEMFU) goal & objectives. Goal: Emergency medicine (EM) residents, through further supervised pediatric experience, reflection, knowledge development, and understanding of the acute presentation and longitudinal course of pediatric illness and injury, will be able and competent to provide evidence based excellent care to ill and injured children.

Objective	Curriculum components	ACGME core competencies
EM residents will demonstrate and apply developmentally appropriate practice in pediatrics, developing skills in the approach to children of a variety of ages and developmental stages	- ED-based clinical care - Direct observation - Reflection	PC, PBLI, ICS, P
EM residents will demonstrate application of patient- and family-centered practice, recognizing and integrating the importance of social and family factors in pediatric care	- ED-based clinical care - Direct observation	PC, PBLI, ICS, P
EM residents will continue to develop sound clinical reasoning, and discuss and provide support for appropriate evidence-based management and disposition of acutely ill and injured children	- ED-based clinical care - Patient follow-up - Ward rounds - Educational conference -Core content reading	PC, MK, PBLI, SBP, ICS, P
EM residents will reflect upon their professional identities as emergency physicians, able to collaborate with colleagues from many disciplines, and secure in their roles in the continuum of medical care of patients and families	- ED-based clinical care - Direct observation - Reflection	PBLI, ICS, P
EM residents will appraise and critique – through patient outcomes, reading, discussion, and written analysis – an array of approaches to pediatric complaints and conditions.	- Patient follow-up - Core content reading - Case report/literature review	MK, PBLI, SBP

*PC,* patient care; *MK,* medical knowledge; *PBLI,* practice-based learning/improvement; *SBP,* systems-based practice; *ICS,* interpersonal/communication skills; *P*, professionalism, *ACGME,* Accreditation Council for Graduate Medical Education

**Table 2:** Pediatric emergency medicine with follow-up: eight specific educational interventions.

Emergency department (ED) based clinical care (5 weekly shifts in the pediatric ED), supervised by pediatric emergency medicine (PEM) subspecialists This component comprises the core clinical experience, and is the basis on which the situated learning curriculum rests. Based *in the ED*, it characterizes situated learning for emergency physicians (EPs) in training. Follow-up on all admitted patients (visiting patients at the bedside, reading daily progress notes, follow-up on test results, interaction with inpatient teams). Following the course of admitted patients, EM residents learn to anticipate the progression of pediatric illness and the rationale for therapies utilized. An important skill, practicing EPs frequently perform patient follow-up as a form of self-directed education, continuous quality improvement and professional satisfaction. Telephone follow up on patients discharged from the ED (minimum 2 patients/week). A log is kept, including pertinent follow-up details. Through telephone follow up with discharged patients, EM residents identify opportunities for improvement, and incorporate this feedback into future practice. Weekly “ward rounds” with PEM faculty, seeing inpatients at the bedside, discussing the presentation, clinical or psychosocial findings, diagnoses, treatment and/or outcomes EM residents identify the effects of illness or injury on patients/families, incorporating feedback into future practice. Faculty use modeling, coaching and scaffolding techniques to externalize thought processes, encouraging discussion and reflection. Attendance at a weekly educational conference This conference focuses on clinical and systems issues, moderated by pediatric hospitalist faculty. EM residents participate to understand issues that affect patients and families whose illness experience includes the ED. Core content reading list and completion of 10 online modules Each EM resident works through a core set of literature and asynchronous online modules^23^ (used with authors’ permission), accompanied by guiding objectives and serving as the basis for teaching discussions and further self-directed learning. Direct observation session: Once per month, for 2–3 hours, the EM resident is directly observed in their ED interactions with patients, families, and other providers. EM faculty performing this observation focus on coaching, providing formative feedback using a standardized tool based on entrustable professional activities. A self-reflection exercise is built into this feedback, encouraging the learner to reflect on his or her ongoing professional identity formation Case-based written report: The resident identifies a clinical question or case encounter, and writes a brief review of the literature to illustrate important PEM concepts. This exercise reinforces the importance of lifelong, self-directed learning. Faculty provide feedback, and cases and discussions are shared via a moderated online blog with an associated discussion forum for post-publication peer review.
