# Supplementary material for: A Novel Curriculum to Optimize Emergency Medicine Residents’ Exposure to Pediatrics
Source: West J Emerg Med. 2016 Nov 15;18(1):14–9. doi: 10.5811/westjem.2016.10.31248 (PMC5226749; doi:10.5811/westjem.2016.10.31248)
Supplement: Supplementary file 1 [file wjem-18-14-s001.docx]

Inputs & Resources

Impact

Activities

Outcomes

Outputs

To accomplish our activities, we will need the following:

To address this educational need, our learners will perform the following activities:

Faculty time (Course director, clinical faculty)

Resident time

Program support

Clinical materiel (ED & inpatient)

Adminis-trative support

Once we complete these activities, we expect to produce the following evidence of program delivery:

Once complete, we expect that program delivery will lead to the following changes:

We expect that, once completed, these outcomes will result in the following long-term impact:

Provide a focused, month-long pediatric ED experience

Inpatient & outpatient follow-up, weekly attending rounds

Reading & asynchronous modules

Self-directed learning & writing project

Attendance at weekly educational conference

1 EM resident per month (12 per year)

5 clinical shifts/week

1 direct observation session per month

Once weekly ward rounds

1 monthly work product based on a clinical question or illustrative case discussion

Case log of all admitted patients

Case log of follow-up on discharged patients

Learners will:

- Maintain excellent ITE scores

- Develop competence in developmentally appropriate, patient- and family-centered care

- Develop competence in management of pediatric illness and injury

- Provide improved care for children throughout their residencies

- Develop a mature professional identity, including recognition of children as a primary population/ responsibility of emergency physicians

Sustained excellence in care for ill and injured children in US EDs

**Assumptions**:

To excel, emergency physicians must have an understanding of the natural course of illness and be able to anticipate the needs of patients who are admitted to the hospital.

After losing a dedicated inpatient pediatric medicine rotation, the opportunity to gain these experiences and exposures may have been lost, along with other less tangible learning opportunities, such as collegial bonding and a basic understanding of the systems of inpatient care.

An alternate, ED-based educational experience may allow emergency medicine trainees a more high yield opportunity to gain these needed skills, knowledge, attitudes and experiences.

**Appendix.** *Logic model.* A logic model was developed to guide the specific education strategies employed in the PEMFU rotation and to direct program evaluation.
